# Mechanical-acoustic-optical response properties in uniaxial compression failure of rock masses with different dissolution degrees

**DOI:** 10.1371/journal.pone.0360540

**Published:** 2026-10-13

**Authors:** Wenyu Luo, Jiaqi Guo, Fan Chen, Tongxin Chen, Yipeng Wang

**Affiliations:** School of Civil Engineering, Henan Polytechnic University, Jiaozuo, Henan, China; The University of Hong Kong, HONG KONG

## Abstract

To reveal the effects of dissolution cavities on the mechanical properties and failure mechanisms of surrounding rock, uniaxial compression tests were conducted on rock-like specimens with dissolution degrees of 0%, 5%, 10%, 15%, and 20%. A mechanical-acoustic-optical integrated system combining acoustic emission (AE) and digital image correlation (DIC) was used to characterize mechanical response, AE activity, strain field evolution, and failure behavior. The results indicate that with increasing dissolution degree, compressive strength, elastic modulus, peak axial strain, and deformation modulus decreased by 36.45%, 27.01%, 17.25%, and 41.28%, respectively. Post-peak failure gradually changed from rapid coalescence of a single dominant crack to progressive failure controlled by multiple defects. AE activity shifted from concentrated release near peak strength to continuous release throughout loading, accompanied by earlier crack initiation and an expanded pre-peak damage interval. Crack activity evolved from small scale microcracking to the propagation and coalescence of larger scale cracks. Combined AE and DIC results further showed a transition from axial tensile splitting failure to progressive mixed tensile-shear failure with multiple shear strain concentration zones and coordinated crack coalescence. These findings provide experimental evidence for stability assessment and disaster mitigation of dissolution voided surrounding rock in karst underground engineering.

## 1. Introduction

Karst landscapes account for approximately one fifth of the global terrestrial surface. Under different burial depth conditions, rock masses may develop karst structures with markedly different degrees of dissolution development [1]. In addition to internal structural planes, their mechanical properties are strongly affected by numerous dissolution cavities and fissures, resulting in highly complex and variable mechanical behavior of dissolved rock masses. To date, extensive research has been conducted on the mechanical properties of conventional rock masses containing joints and fractures [2], whereas systematic investigations into the mechanical evolution of rock masses containing dissolution cavities remain relatively limited. With the continued expansion of underground engineering projects such as tunnels, engineering problems associated with the stability of dissolution voided tunnel surrounding rock and slopes have become increasingly prominent. Therefore, elucidating the mechanisms by which karst cavities affect the mechanical response of rock masses is of considerable importance [3].

Numerous studies have investigated the mechanical properties, crack propagation, and damage and failure mechanisms of dissolved rock masses. In terms of theoretical analysis, Xi et al. [4] proposed a numerical modeling method for rock masses containing dissolution cavities based on a stochastic medium mathematical model. She et al. [5] conducted dissolution experiments and revealed the controlling effect of karst pore structures on the progressive dissolution process of rock masses. Li et al. [6] combined hydrochemical analysis with fractal theory to investigate the relationship between dissolution morphology and hydrochemical characteristics. Shang et al. [7] classified different dissolution zones in karst regions according to the degree of karst development and variations in dissolution intensity. Hu et al. [8] combined power function fitting with the Hoek-Brown strength criterion to establish quantitative relationships between porosity and the mechanical parameters of dissolved rock masses. In terms of laboratory experiments, Palchik [9] investigated the conventional mechanical parameters of limestones from different karst regions and demonstrated that heterogeneous structures affect the relationship between the elastic modulus and compressive strength of carbonate rocks. Guo et al. [10] and Chen et al. [11] investigated the mechanical characteristics, energy evolution, and AE responses of karst limestone under different water content conditions. Huang et al. [12] analyzed the effects of different rock mass structures and bank slope configurations on the deterioration characteristics and mechanical properties of shallow rock masses within the water level fluctuation zone of karst slopes. Wang et al. [13] investigated the microcrack evolution mechanisms of limestone containing dissolution fissures with different geometries based on acoustic characteristics obtained from uniaxial compression tests. Ma et al. [14] conducted uniaxial compression tests on rock-like specimens containing spherical defects and analyzed the effects of cavity size and spatial position on the mechanical parameters and stress distributions of the specimens. Cai et al. [15] further investigated the effects of dissolution on the mechanical parameters of rock masses with different dissolution degrees. In terms of numerical simulations, Liu et al. [16] constructed dissolved rock mass models containing vertical elliptical dissolution cavities and investigated the stability of karst rock foundations. Liu et al. [17,18] employed numerical methods to analyze the mechanical responses and failure characteristics of marlite at different dissolution stages. Yu et al. [19] constructed numerical models of dissolved rock masses using the Monte Carlo method and investigated the influence of karstification ratio on rock mass mechanical properties. Yu et al. [20] investigated the contact force chain characteristics, stress-strain behavior, and failure characteristics of dissolved rock masses under uniaxial compression. Karl et al. [21] combined probabilistic analysis with finite element modeling to investigate the effects of the spatial distribution of karst cavities on tunnel lining stability. Miao et al. [22] employed ABAQUS to investigate the mechanical characteristics and damage evolution mechanisms of dissolved sandstone under uniaxial compression. Zhang et al. [23], Leng et al. [24], and Shi et al. [25] constructed different types of dissolved rock mass models using discrete element methods and revealed the effects of dissolution structures on rock mass strength and failure modes. Xia et al. [26] further analyzed the effects of randomly distributed karst cavity groups on tunnel stability based on continuum damage mechanics. These studies have revealed, from different perspectives, the controlling effects of karst development on strength degradation, crack propagation paths, and failure modes of rock masses. Nevertheless, existing studies have predominantly focused on numerical simulations or single monitoring parameters to investigate the effects of individual large karst cavities. The intrinsic relationships among mechanical parameter deterioration, AE responses, and surface strain field evolution during the uniaxial compression of rock masses containing numerous small and medium scale cavities with different dissolution degrees remain insufficiently understood experimentally. Therefore, synchronous AE and DIC monitoring is required to further investigate the damage evolution and failure behavior of rock-like specimens with different dissolution degrees.

Accordingly, uniaxial compression tests were conducted on artificially prepared rock-like specimens with dissolution degrees of 0%, 5%, 10%, 15%, and 20%, combined with synchronous AE and DIC monitoring. Different from previous studies focusing primarily on individual cavities or single monitoring indicators, the present study systematically investigates the progressive deterioration and failure evolution of rock-like specimens over a controlled dissolution degree gradient. A mechanical-acoustic-optical integrated approach was employed to establish the relationships among mechanical deterioration, AE activity and crack scale evolution, DIC strain localization, and macroscopic failure modes. On this basis, the transition of the failure mechanism from localized tensile splitting to progressive multi defect controlled mixed tensile-shear failure was clarified. These findings provide experimental evidence for understanding the damage evolution of dissolved rock masses and for the stability assessment of dissolution voided surrounding rock in karst underground engineering.

## 2. Specimen preparation and testing methods

### 2.1. Specimen preparation

To simulate the internal cavity structures of dissolved rock masses, cylindrical rock-like specimens with dissolution defects, measuring 50 mm in diameter and 100 mm in height, were prepared using the pre-embedded ice ball melting method. This approach helps preserve the overall integrity of the cast specimens and facilitates the preparation of multiple enclosed internal cavities. During specimen casting, spherical ice balls with a diameter of 10 mm were embedded within the specimens. After initial setting, the ice balls melted naturally, thereby forming approximately spherical internal cavities. According to the prescribed dissolution degrees, five groups of specimens with dissolution degrees of 0%, 5%, 10%, 15%, and 20% were prepared by controlling the total volume of the prefabricated cavities. The specimen with a dissolution degree of 0% was used as the intact reference group. The remaining dissolution degrees of 5%, 10%, 15%, and 20% were selected at equal 5% intervals to establish a progressive gradient of internal cavity development. Within the experimental range considered in this study, 5%-10% represent relatively low to moderate dissolution conditions, whereas 15%-20% represent relatively high dissolution conditions. In this study, the dissolution degree was defined as the volume fraction of the internal cavities and calculated as:


$$\begin{array}{c} D=\frac{V_{c}}{V_{s}}\times 100\% \end{array}$$ (1)

where *D* is the dissolution degree, *V*_*c*_ is the total volume of the prefabricated cavities, and *V*_*s*_ is the total volume of the cylindrical specimen. The specimen with a dissolution degree of 0% contained no prefabricated cavities and was used as the intact reference group. By adjusting the total cavity volume, different dissolution degrees were obtained to represent different levels of structural deterioration in dissolved rock masses.

To ensure the integrity and spatial stability of the prefabricated ice balls during specimen preparation, spherical ice balls with a diameter of 10 mm were prepared in advance using molds. Broken or deformed ice balls were removed, and the remaining ice balls were stored at a low temperature to prevent premature melting. A layered casting procedure was adopted during specimen preparation. The thickness of each casting layer and the positions of the ice balls were strictly controlled, and appropriate radial and axial spacing was maintained between adjacent ice balls. By controlling the layer thickness, number of ice balls, and spatial arrangement, a relatively uniform distribution of internal cavities was achieved for specimens with different dissolution degrees, thereby improving the reliability and repeatability of the test results. The specimen preparation procedure is illustrated in Fig 1.

**Fig 1:**
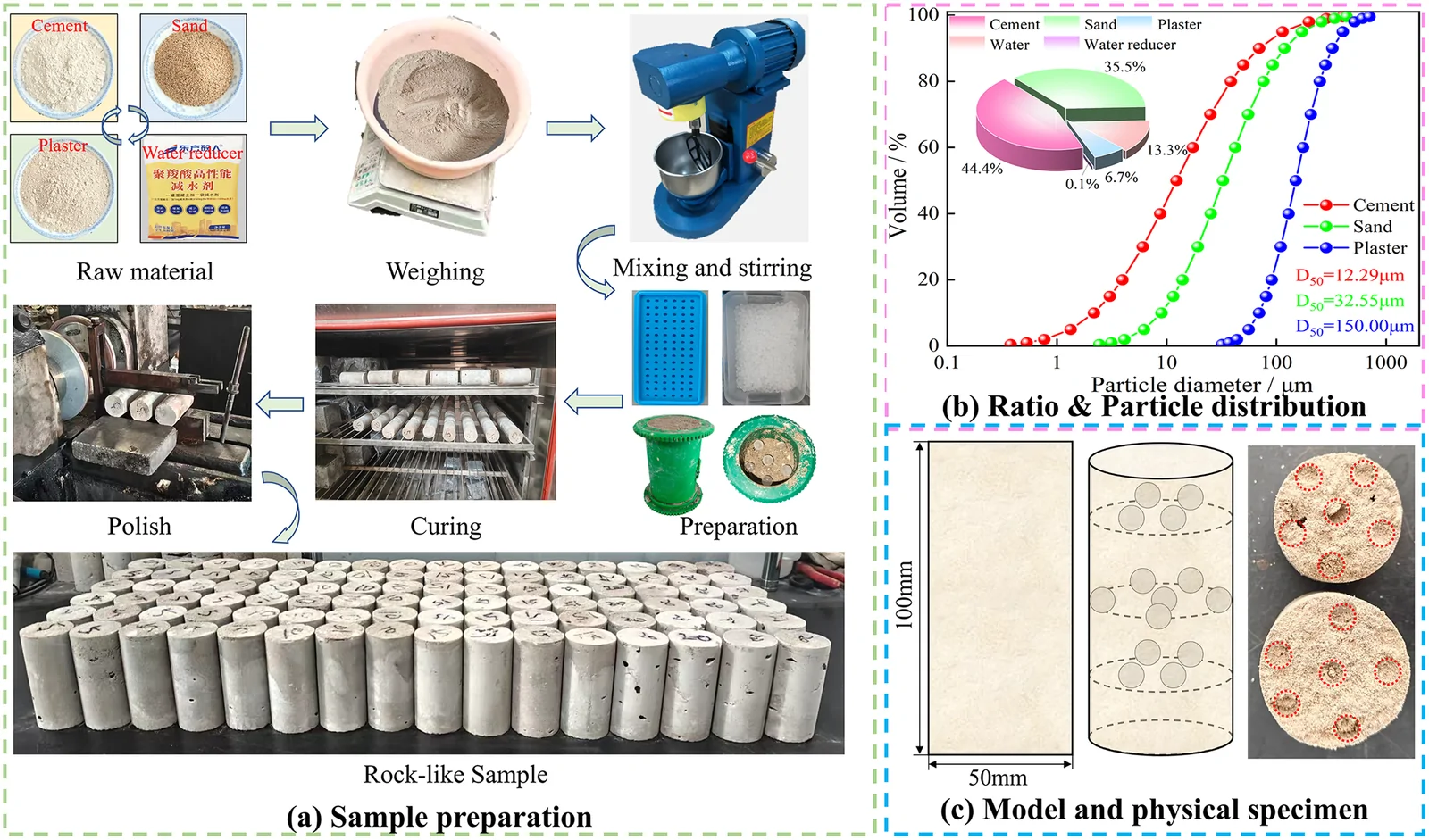
Specimen preparation procedure.

The raw materials consisted of P.O. 42.5 ordinary Portland cement, fine river sand, high strength gypsum, polycarboxylate superplasticizer, and purified water. Based on mix proportion tests, the mass ratio of cement, sand, gypsum, superplasticizer, and water was determined as 1:0.8:0.15:0.003:0.3. After casting, the specimens were left undisturbed for 24h before demolding. The embedded ice balls melted naturally to form the designed internal cavity structures. The demolded specimens were subsequently cured for 28d at a temperature of 40°C and a relative humidity of 90%. After curing, the specimens were visually inspected and dimensionally examined under ambient humidity conditions. Specimens exhibiting visible cracks, chipped edges, or uneven end surfaces were discarded. Both end surfaces of the qualified specimens were carefully ground to satisfy the requirements for flatness and parallelism in uniaxial compression tests. Before all specimens were tested under the natural moisture condition, a random speckle pattern was prepared on the observation surface for DIC measurement. Surface dust was first removed, after which a uniform layer of white matte primer was applied. Following drying, randomly distributed black speckles were produced on the white background using a black marker to form a high contrast pattern covering the observation region. The specimens were then left for 24h before the synchronous uniaxial compression, AE, and DIC tests were conducted.

### 2.2. Experimental apparatus and procedure

The experimental system consisted of a rock mechanics loading system, an acoustic emission monitoring system, and a digital image correlation monitoring system, as shown in Fig 2. Uniaxial compression tests were conducted on individual specimens with dissolution rates of 0%, 5%, 10%, 15%, and 20%, respectively. During testing, the three systems were operated synchronously to acquire the mechanical, acoustic, and optical information throughout the deformation and failure process. The mechanical loading system comprised an RMT-150B electro hydraulic servo controlled testing machine, which was employed to obtain the mechanical responses of rock-like specimens with different dissolution degrees under uniaxial compression. Axial displacement controlled loading was adopted at a constant loading rate of 0.005 mm/s. AE monitoring was performed using a Micro-II acoustic emission testing and analysis system to capture the elastic wave signals generated during microcrack initiation, propagation, and coalescence within the specimens. Four AE sensors were symmetrically mounted on both sides of each specimen. An acoustic coupling agent was applied between the sensors and the specimen surface to ensure satisfactory signal transmission. The AE threshold, preamplifier gain, and sampling frequency were set to 40 dB, 40 dB, and 5 MHz, respectively. The DIC monitoring system was used to record the evolution of the surface strain field and localized deformation, thereby revealing the controlling effect of dissolution cavities on the damage evolution and failure process of the rock-like specimens at different scales. The high speed camera had a maximum resolution of 4096 × 2160 pixels and a maximum acquisition frequency of 60 Hz. Before testing, a random speckle pattern was prepared on the observation surface of each specimen. The camera position, focal length, and illumination intensity were then adjusted to ensure that the acquired speckle images exhibited high contrast, stable illumination, and satisfactory clarity. The images acquired before and during loading were processed using Vic-2D software to obtain the evolution of the surface strain and displacement fields.

**Fig 2:**
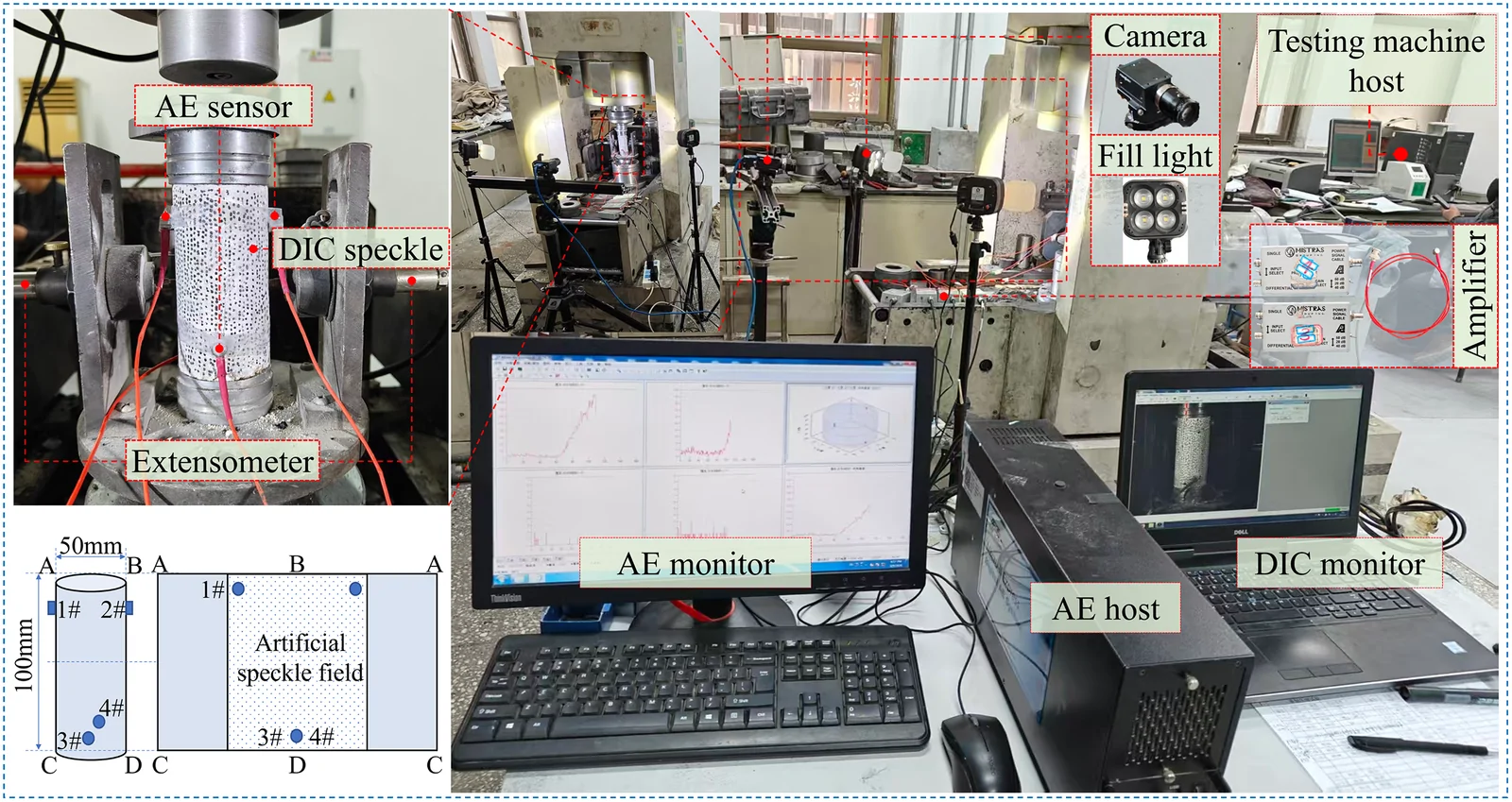
Specimen and monitoring systems.

To ensure the comparability of the multi source monitoring data, loading time was adopted as the common reference for synchronizing the stress-strain curves, AE parameters, and DIC images. The mechanical, acoustic, and optical monitoring results were mutually corroborated, enabling a relatively comprehensive characterization of the mechanical deterioration, damage evolution, and failure mechanism of rock-like specimens induced by dissolution cavities.

## 3. Experimental results and analysis

### 3.1. Mechanical characteristics

#### 3.1.1. Stress-strain curves.

Fig 3 presents the stress-strain curves of rock-like specimens with different dissolution degrees under uniaxial compression. All specimen groups exhibit pronounced stage dependent characteristics, and their deformation and failure processes can be divided into a compaction stage, a near linear elastic stage, a stable crack propagation stage, and a post-peak failure stage. During the initial loading stage, the curves increase slowly in a nonlinear manner and exhibit relatively small slopes. At this stage, the primary pores and microdefects around the cavity boundaries gradually close, and specimen deformation is dominated by compaction. As the stress increases, the curves enter a near linear stage, during which the internal structure gradually stabilizes and stress and strain increase almost synchronously, indicating predominantly elastic deformation. When the applied stress approaches the peak strength, the curves gradually deviate from linearity. Cracks begin to initiate and propagate around the cavity boundaries and within locally weakened regions, marking the onset of damage development. After the peak strength is reached, the stress decreases, while the cracks evolve from dispersed propagation to progressive coalescence and eventually form macroscopic fracture surfaces.

**Fig 3:**
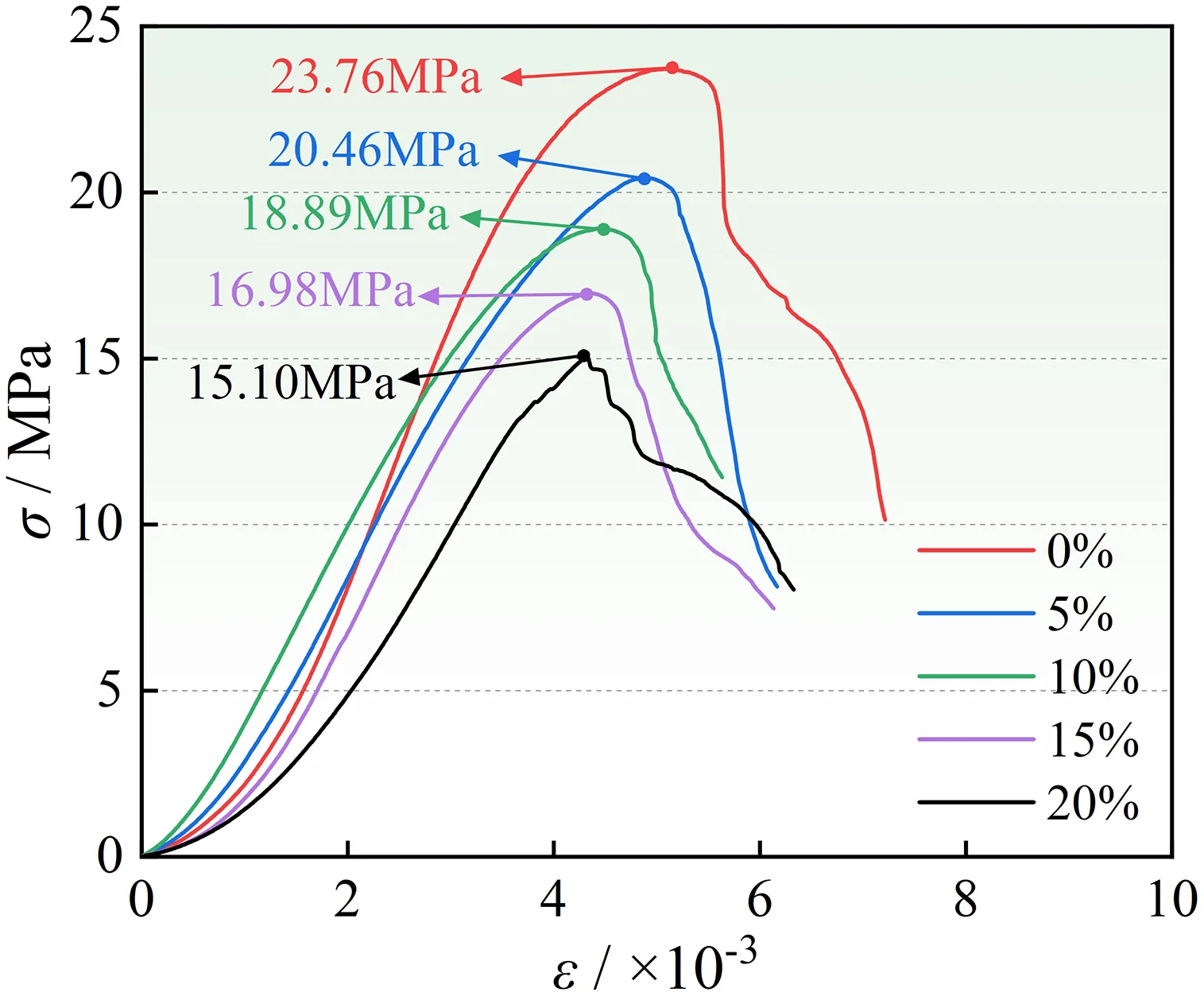
Stress-strain curves of specimens with different dissolution degrees.

As the dissolution degree increases, the peak strength of the specimens decreases continuously. The peak strength of the intact specimens is 23.76 MPa. When the dissolution degree increases to 5%, 10%, 15%, and 20%, the peak strength decreases to 20.46, 18.89, 16.98, and 15.10 MPa, respectively, with the reduction becoming progressively more pronounced. An increase in the number of cavities segments the effective load bearing skeleton and reduces the effective load bearing area. Meanwhile, stress concentration around the cavity boundaries promotes earlier crack initiation and propagation at lower stress levels. Consequently, specimens with higher dissolution degrees enter the nonlinear deformation stage earlier and exhibit substantially reduced peak strength and pre-peak load bearing capacity.

In terms of curve morphology, the intact specimens exhibit relatively steep pre-peak curves, a distinct near linear elastic stage, satisfactory structural integrity, and strong resistance to deformation. After the peak, the stress decreases rapidly and exhibits slight fluctuations, indicating that local blocks retain a short term load bearing capacity and undergo frictional adjustment after the formation of the main crack. With increasing dissolution degree, the pre-peak curves become progressively gentler, the near linear elastic stage shortens, and the nonlinear characteristics become increasingly pronounced. This behavior is particularly evident for the specimens with dissolution degrees of 15% and 20%, whose curves exhibit obvious curvature at relatively low stress levels. Under these conditions, the internal cavities gradually dominate the deformation and failure processes, while crack propagation is increasingly controlled by the spatial distribution and interaction of the cavities.

Increasing dissolution degree also alters the post-peak failure response of the specimens. Specimens with low dissolution degrees exhibit a pronounced post-peak stress drop and relatively strong brittle failure characteristics. By contrast, the post-peak curves of specimens with high dissolution degrees decrease more gradually, and a certain residual load bearing capacity is retained during the residual stage. This behavior is likely associated with the progressive propagation of multiple cracks induced by the internal cavities. In intact specimens and specimens with low dissolution degrees, the number of internal defects is relatively small, allowing substantial elastic energy to accumulate before the peak. Once the main crack coalesces, the load bearing structure fails rapidly, resulting in a concentrated post-peak stress drop. For specimens with high dissolution degrees, the cavities and microdefects participate in deformation at an earlier loading stage. Cracks successively initiate, propagate, and coalesce within multiple localized regions, resulting in substantial pre-peak damage. Accordingly, the post-peak failure process gradually transforms from rapid coalescence of a single dominant crack to progressive failure controlled by multiple defects.

#### 3.1.2. Deformation parameters.

Fig 4 presents the variations in peak strength, elastic modulus, peak axial strain, and deformation modulus of the rock-like specimens with different dissolution degrees under uniaxial compression. As the dissolution degree increases from 0% to 20%, all mechanical parameters exhibit decreasing trends and maintain satisfactory linear relationships with dissolution degree, with correlation coefficients greater than 0.95. The different mechanical parameters show different sensitivities to dissolution degree. The deformation modulus exhibits the largest reduction, followed by the peak strength, whereas the peak axial strain shows the smallest relative reduction.

**Fig 4:**
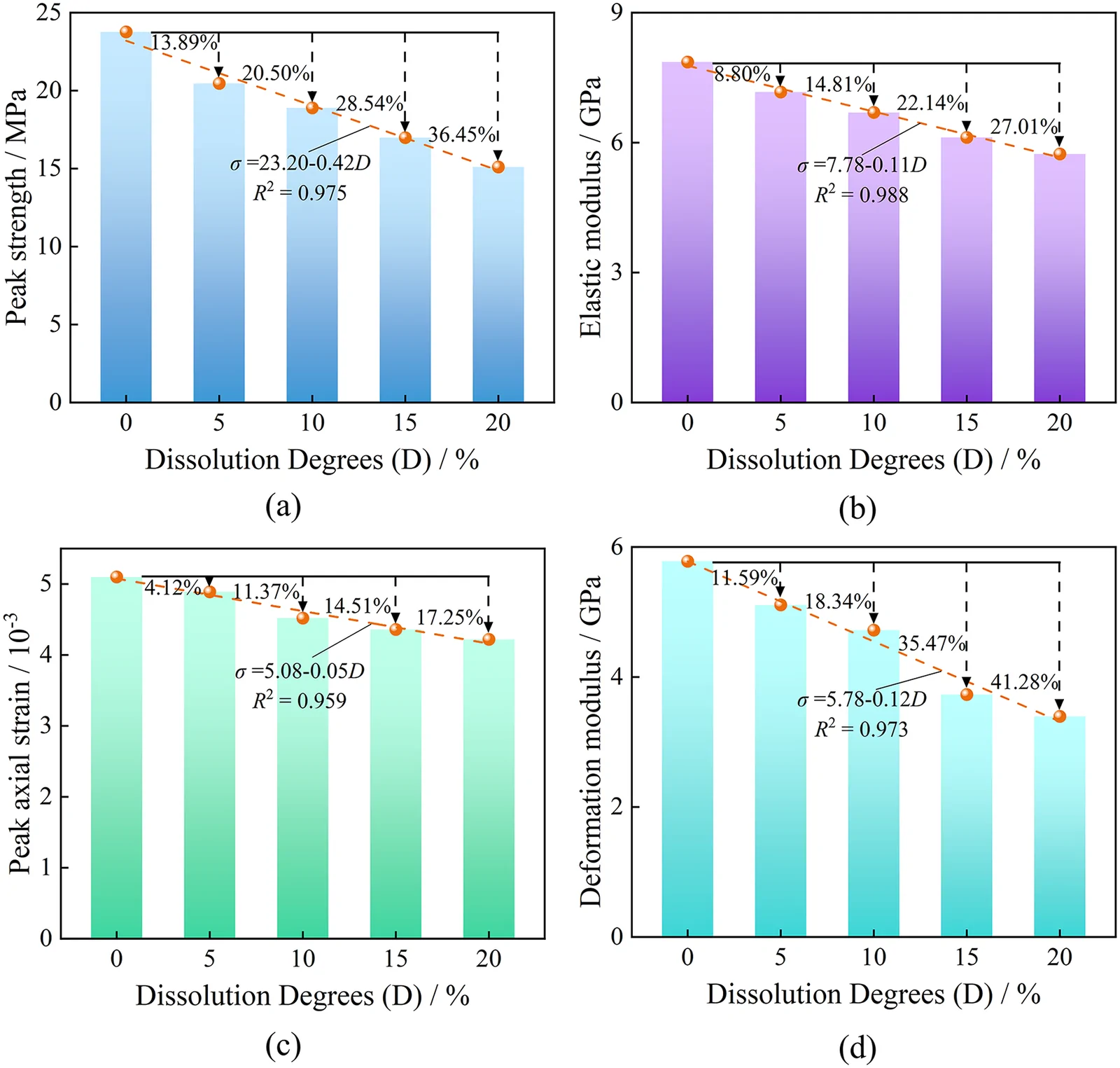
Relationships between the deformation parameters and dissolution degree. (a) peak strength, (b) elastic modulus, (c) peak axial strain, (d) deformation modulus.

As shown in Fig 4(a), the peak strength decreases approximately linearly with increasing dissolution degree. It decreases from 23.76 MPa at a dissolution degree of 0% to 15.10 MPa at a dissolution degree of 20%, corresponding to a reduction of 36.45%. This reduction is primarily attributed to the increase in cavity volume fraction, which decreases the effective load bearing area and disrupts the uniform transmission of the applied load. Meanwhile, stress concentration around the cavities promotes earlier crack initiation and propagation toward adjacent cavities. Consequently, specimens with high dissolution degrees undergo substantial localized damage before reaching the peak strength, resulting in a pronounced reduction in macroscopic peak strength. As shown in Fig 4(b), the elastic modulus also decreases with increasing dissolution degree. When the dissolution degree increases from 0% to 20%, the elastic modulus decreases by 27.01%. With increasing dis-solution degree, the number of cavities increases, reducing the continuity of the specimen matrix and weakening the stress transfer paths between particles and cemented structures. This leads to a decrease in the slope of the pre-peak near linear elastic stage and consequently weakens the initial stiffness and deformation resistance of the specimens.

As shown in Fig 4(c), the peak axial strain generally decreases with increasing dissolution degree. The peak axial strain of the intact specimens is approximately 5.08 × 10^−3^. When the dissolution degree increases to 20%, the peak axial strain decreases to approximately 4.20 × 10^−3^, corresponding to a reduction of 17.25%. Compared with the peak strength and elastic modulus, the decrease in peak axial strain is relatively small. Nevertheless, this variation indicates that dissolution defects reduce the effective deformation capacity that can be accumulated before the peak strength is reached. As shown in Fig 4(d), the deformation modulus is more sensitive to dissolution degree than the other mechanical parameters. As the dissolution degree increases from 0% to 20%, the deformation modulus decreases from 5.78GPa to 3.39GPa, corresponding to a reduction of 41.28%. This reduction is greater than those observed in the peak strength, elastic modulus, and peak axial strain. The cavity defects not only reduce the stiffness during the near linear elastic stage but also promote earlier crack activity during the initial loading and pre-peak nonlinear stages, resulting in a rapid deterioration of the overall deformation resistance. This effect is particularly pronounced in the specimens with dissolution degrees of 15% and 20%, in which localized damage zones readily expand and coalesce, thereby causing a substantial re-duction in the deformation modulus.

### 3.2. Acoustic characteristics

#### 3.2.1. AE ring count characteristics.

Fig 5 presents the AE ring counts, cumulative AE ring counts, stress evolution, and DIC axial strain fields at representative loading moments for specimens with different dissolution degrees during uniaxial compression. Overall, the AE response corresponds closely to the stress time curves and exhibits pronounced stage dependent characteristics. Based on the combined evolution of stress, AE ring counts, and DIC axial strain fields, the specimen damage process can be divided into a compaction stage, a near linear elastic stage, a stable crack propagation stage, and a post-peak failure stage. All specimens exhibit an evolution from weak AE activity through intermittent release and concentrated enhancement to sustained post-peak release. However, the onset time, degree of concentration, and cumulative growth pattern of AE ring counts vary considerably with dissolution degree.

**Fig 5:**
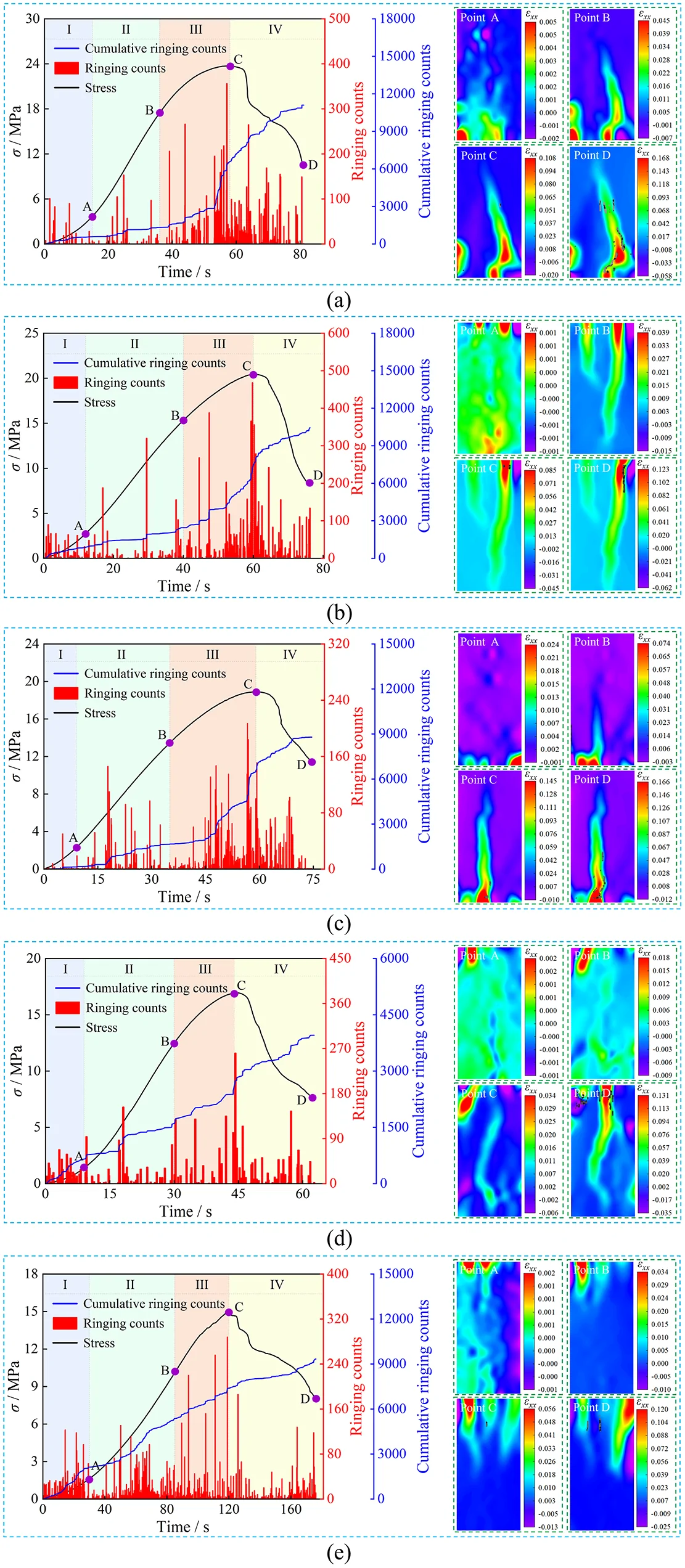
AE ring count characteristics of specimens with different dissolution degrees. (a) 0%, (b) 5%, (c) 10%, (d) 15%, (e) 20%.

For the intact specimen, AE activity remains weak during the initial loading stage, and the cumulative AE ring count increases slowly, owing to the relatively intact internal structure and high crack initiation threshold. As the stress approaches the peak strength, the AE ring counts increase rapidly. Meanwhile, the DIC axial strain field shows that a localized deformation band forms and coalesces during the later loading stage, indicating typical brittle failure. When the dissolution degree increases to 5% and 10%, AE activity occurs earlier, and the cumulative AE ring count increases markedly before the peak. Stress concentration induced by the cavities promotes earlier crack initiation and propagation around the cavity boundaries, while the axial strain fields reveal that localized deformation zones have already developed during the pre-peak stage. Therefore, the damage evolution changes from concentrated release near the peak to progressive development before the peak. At a dissolution degree of 15%, AE activity becomes pronounced during the early loading stage, and the cumulative AE ring count increases continuously. Cracks initiate at multiple locations and gradually coalesce, while the axial strain field exhibits several localized strain concentration zones. The failure process consequently transforms from one controlled by a single dominant crack to the coordinated propagation of cracks induced by multiple defects. At a dissolution degree of 20%, AE activity remains continuously active from the initial loading stage, and the AE ring counts are released in a more dispersed manner. The abrupt AE response near the peak becomes less pronounced, the axial strain distribution becomes increasingly heterogeneous, and the crack paths exhibit multiple branches. These characteristics indicate that the failure process of specimens with high dissolution degrees is dominated by continuous damage accumulation. Overall, increasing dissolution degree transforms AE activity from concentrated release near the peak to continuous release throughout the loading process. Crack initiation occurs earlier, crack propagation becomes more dispersed, and the failure behavior gradually changes from concentrated brittle failure to progressive damage and failure.

#### 3.2.2. Peak frequency characteristics.

The AE peak frequency can be used to characterize the scale of internal crack activity and the fracture process of the specimens. High-frequency signals are generally associated with microcrack initiation and localized propagation, intermediate frequency signals correspond to crack propagation, coalescence, and local frictional adjustment, whereas low-frequency signals are related to large scale crack coalescence, the formation of macroscopic fracture surfaces, and frictional sliding along crack surfaces. To investigate the crack scale evolution of rock-like specimens with different dissolution degrees during uniaxial compression, the peak frequency was divided into three bands based on the classification adopted in previous rock-fracture AE studies [27]: low frequency (LF, 0–115 kHz), intermediate frequency (IF, 116–280 kHz), and high frequency (HF, > 280 kHz). The measured AE signals in the present tests were distributed across all three frequency bands and exhibited systematic changes with increasing dissolution degree, to provides a consistent framework for comparing the relative evolution of AE frequency characteristics among different dissolution degrees. The proportions of AE events within the three frequency bands are presented in Fig 6. All specimens generated multi frequency AE events during loading, indicating that crack evolution underwent a continuous transition from microcrack initiation through propagation to eventual coalescence.

**Fig 6:**
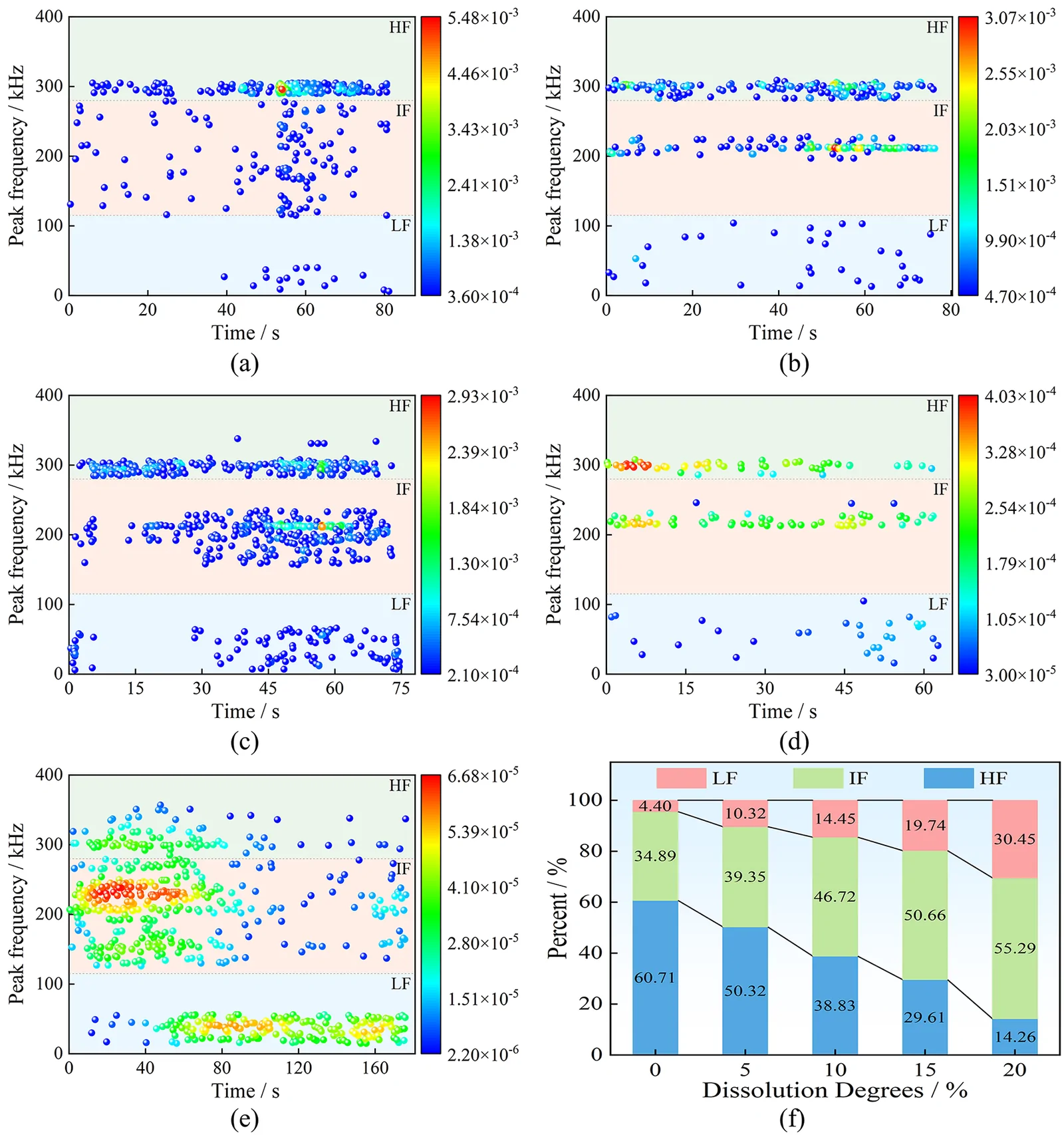
Peak frequency characteristics of specimens with different dissolution degrees. (a) 0%, (b) 5%, (c) 10%, (d) 15%, (e) 20%, (f) proportions of AE events in different peak frequency bands.

For the intact specimen, AE events are dominated by HF signals, which account for approximately 60.71%, whereas IF and LF signals account for 34.89% and 4.40%, respectively. HF signals persist throughout the pre-peak stage, IF signals occur mainly during the middle and later loading stages, and LF signals remain relatively sparse and dispersed. This distribution is attributed to the satisfactory structural continuity of the intact specimen. During the early loading stage, deformation is mainly associated with pore compaction and the closure of pre existing microcracks. Small scale cracks gradually initiate but have not yet developed into coalesced structures, resulting in relatively few large scale fracture events. At a dissolution degree of 5%, the proportion of HF signals decreases to 50.32%, whereas the proportions of IF and LF signals increase to 39.35% and 10.32%, respectively. The introduction of dissolution cavities enhances local stress concentration, causing cracks to initiate at lower stress levels and propagate along the cavity boundaries. Some microcracks consequently evolve into intermediate scale cracks, leading to a pronounced increase in IF and LF signals. At a dissolution degree of 10%, the HF proportion further decreases to 38.83%, while the IF and LF proportions increase to 46.72% and 14.45%, respectively. IF signals become dominant, indicating that crack activity changes from microcrack initiation to propagation and coalescence. The increased number of cavities disrupts the stress transfer paths and generates weakened zones between adjacent cavities, along which cracks more readily deflect, coalesce, and locally penetrate the specimen. When the dissolution degree increases to 15%, the proportion of IF signals rises further to 50.66%, and the proportion of LF signals increases to 19.74%, whereas the HF proportion decreases to 29.61%. The enhanced interaction among the cavities enables cracks to propagate along cavity boundaries and locally weakened regions during the early and middle loading stages. Consequently, intermediate and large scale crack activity becomes increasingly pronounced.

The fracture process exhibits progressive and scale dependent evolution from small cracks to larger cracks, with cracking occurring simultaneously at multiple locations. At a dissolution degree of 20%, the HF proportion decreases substantially to 14.26%, while the IF and LF proportions increase to 55.29% and 30.45%, respectively. IF and LF signals therefore dominate the AE response, indicating that crack activity is primarily characterized by crack coalescence, penetration, and frictional sliding. The dense distribution of cavities considerably weakens the effective load bearing skeleton, allowing cracks to initiate simultaneously within multiple defect regions and subsequently coalesce. The pronounced increase in LF signals corresponds to the intensified formation of macroscopic fracture surfaces. Overall, as the dissolution degree increases from 0% to 20%, the proportion of HF signals decreases continuously, whereas the proportions of IF and LF signals progressively increase. Crack activity consequently transforms from dispersed small scale microcracking to the propagation and coalescence of intermediate and large scale cracks. Dissolution cavities weaken structural continuity, intensify local stress concentration, and shorten the crack coalescence paths, thereby promoting the transition from microscopic damage accumulation to macroscopic coalescence failure.

#### 3.2.3. Failure mode analysis based on RA-AF characteristics.

The RA value is defined as the ratio of the rise time to the amplitude of an AE waveform, whereas the AF value is defined as the ratio of the ring count to the signal duration. Tensile cracks are generally characterized by low RA and high AF values, whereas shear cracks exhibit high RA and low AF values. Therefore, the relative development of tensile and shear cracks during loading and failure can be identified according to the distribution of RA-AF data points on either side of a prescribed classification line. Fig 7 illustrates the characteristic AE waveform parameters and the principle of RA-AF based crack classification. In this study, a slope of 0.2 was adopted for the classification line, expressed as AF = 0.2RA. AE events satisfying AF/RA ≥ 0.2 were classified as tensile cracks, whereas those satisfying AF/RA < 0.2 were classified as shear cracks [28,29].

**Fig 7:**
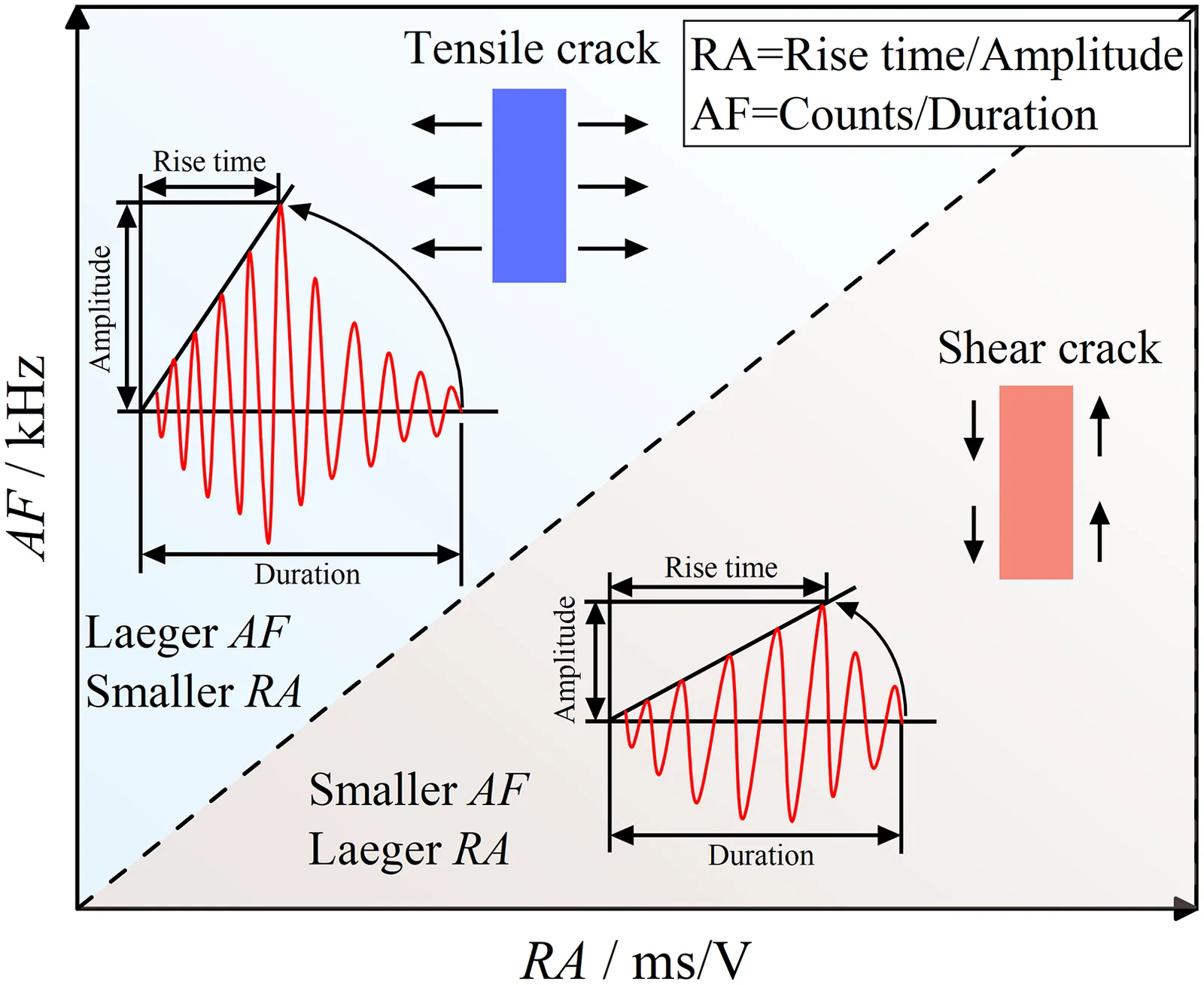
AE waveform parameters and the principle of RA-AF based crack classification.

Fig 8 presents the RA-AF scatter density distributions and the proportions of different cracking modes for specimens with different dissolution degrees. For the intact specimen, tensile and shear cracks account for 61.81% and 38.19% of the AE events, respectively. The RA-AF data points are primarily concentrated in the low RA and high AF region, indicating a tensile dominated failure mode. Owing to the limited number of internal defects and the satisfactory structural continuity of the intact specimen, cracks predominantly initiate and propagate within lateral tensile zones induced by axial compression. With increasing stress, these cracks gradually coalesce to form the main fracture surface, resulting in typical tensile dominated failure. At dissolution degrees of 5% and 10%, the proportions of tensile cracks decrease to 59.68% and 57.03%, respectively, whereas the proportions of shear cracks increase to 40.32% and 42.97%. Although tensile cracks remain dominant, the shear component gradually increases. Stress concentration around the cavity boundaries begins to control the crack propagation paths. Cracks preferentially initiate around the cavity boundaries or locally weakened interfaces and are subsequently affected by geometric constraints and stress redistribution during propagation, thereby inducing local frictional sliding and increasing the proportion of shear cracks. At a dissolution degree of 15%, tensile and shear cracks account for 54.61% and 45.39%, respectively, and the proportions of the two cracking modes become comparable. The RA-AF data points are distributed in both the tensile and shear cracking regions, indicating increasingly complex crack propagation behavior. The increased number of cavities weakens the effective load bearing skeleton. Consequently, cracks not only open around the cavity boundaries but also deflect, coalesce, and slide along the weakened regions between adjacent cavities. The failure mechanism therefore transforms from single tensile crack coalescence to the combined action of multiple cracking mechanisms. When the dissolution degree reaches 20%, the proportion of shear cracks increases to 57.26%, whereas that of tensile cracks decreases to 42.74%, indicating a transition to a shear dominated failure mechanism. The dense distribution of cavities substantially weakens and segments the effective load bearing skeleton, while the reduced spacing between adjacent cavities generates complex stress redistribution zones during loading. Cracks therefore more readily deflect, slide, and coalesce along the weakened zones. Meanwhile, the relative displacement of local blocks becomes more pronounced, resulting in a substantial increase in shear type AE events.

**Fig 8:**
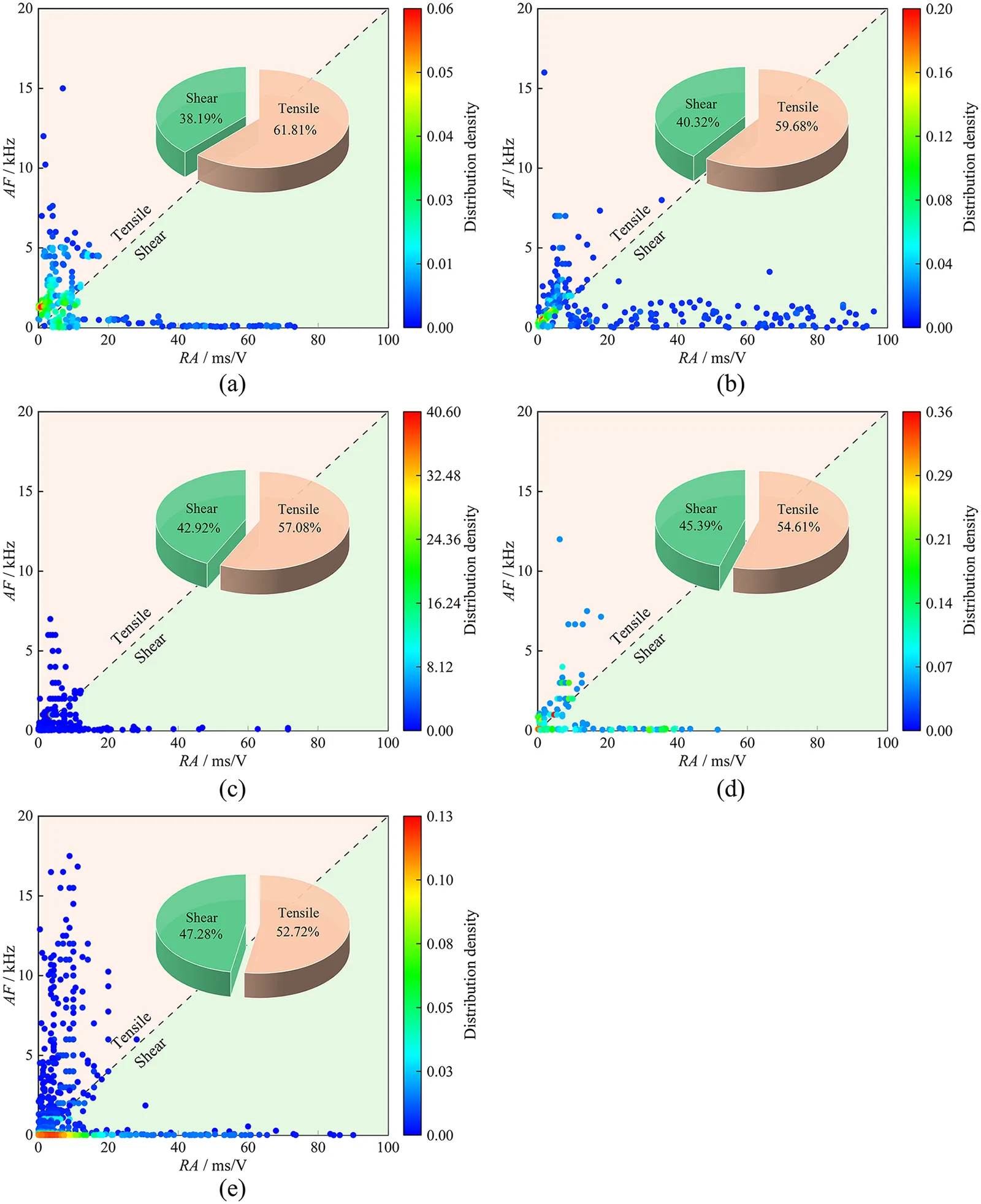
Evolution of cracking modes for specimens with different dissolution degrees. (a) 0%, (b) 5%, (c) 10%, (d) 15%, (e) 20%.

Overall, with increasing dissolution degree, the proportion of tensile cracks decreases continuously, whereas the proportion of shear-cracks progressively increases. The cracking mode evolves from tensile dominated cracking through mixed tensile shear cracking to shear dominated cracking. Increasing dissolution degree enhances the segmentation of the load bearing structure by the cavities, and crack propagation becomes increasingly controlled by cavity boundaries and defect interactions. Accordingly, the failure process gradually transforms from concentrated tensile crack coalescence to complex failure controlled by multiple defects.

### 3.3. Optical characteristics

Fig 9 presents the macroscopic failure morphologies, schematic cracking modes, and DIC shear strain fields of rock-like specimens with different dissolution degrees after uniaxial compression. In the figure, T and S denote tensile and shear cracks, respectively. All specimens develop distinct dominant cracks after failure; however, their crack initiation locations, propagation paths, and distributions of shear strain concentration zones differ considerably. With increasing dissolution degree, the surface cracking pattern gradually changes from relatively concentrated axial coalescence to complex failure characterized by crack initiation at multiple locations, multibranch propagation, and localized shear involvement. Correspondingly, the DIC shear strain field evolves from a single localized band to multiple strain concentration zones.

**Fig 9:**
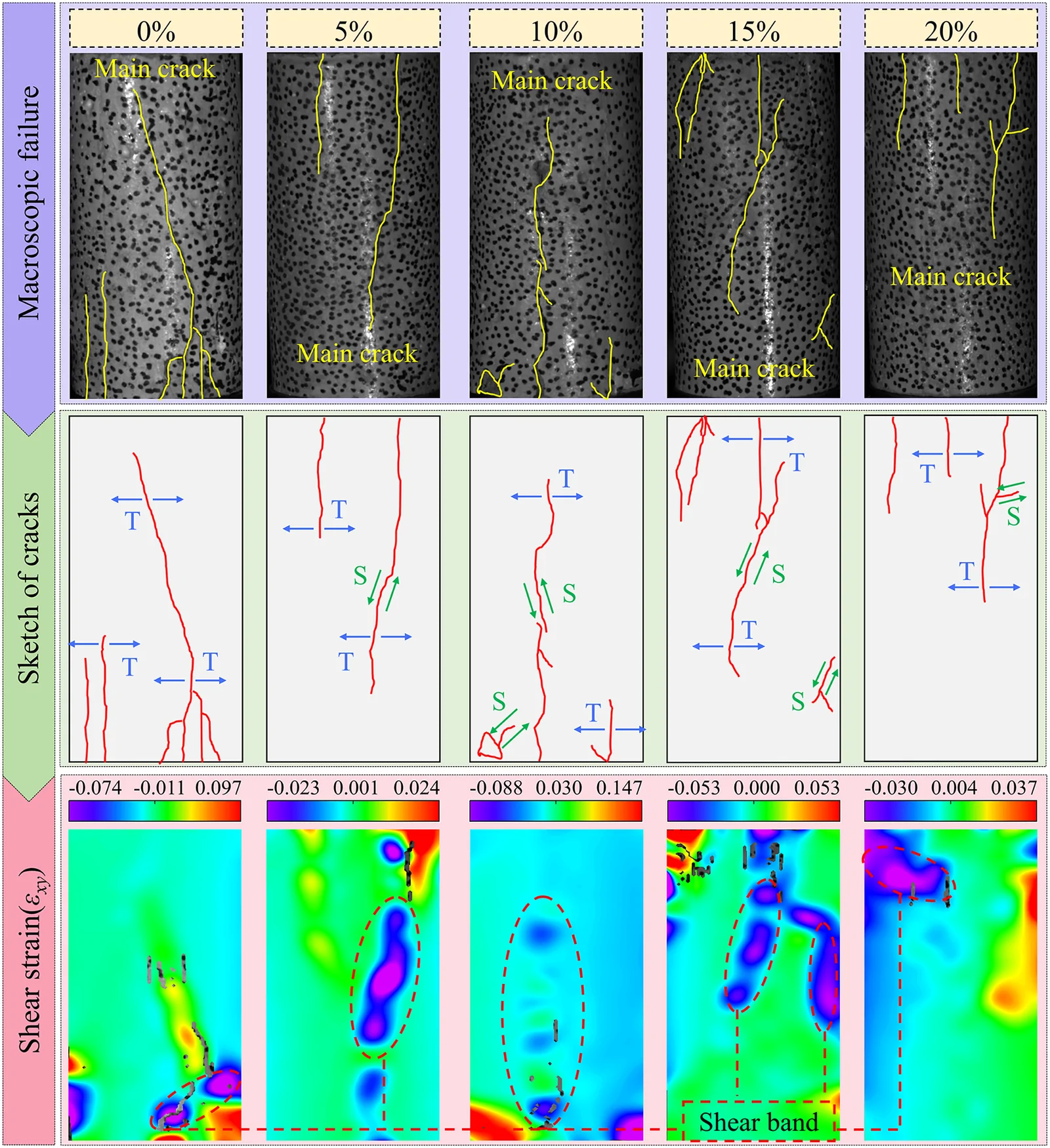
Macroscopic failure morphologies, crack evolution, and shear strain fields of specimens with different dissolution degrees.

For the specimen with a dissolution degree of 0%, an axial dominant crack penetrates through the central portion of the specimen, accompanied by a small number of secondary cracks near the bottom. The failure is dominated by tensile cracking, with only limited local shear cracks. In the DIC shear strain field, the high strain regions are mainly concentrated around the dominant crack and in the lower part of the specimen, and their spatial extent is relatively limited. Owing to the satisfactory continuity of the effective load bearing skeleton, crack propagation is primarily controlled by lateral tensile deformation induced by axial compression. Considerable elastic deformation can therefore accumulate before the peak strength is reached, after which the dominant crack rapidly coalesces, resulting in typical axial tensile splitting failure. At a dissolution degree of 5%, two approximately parallel longitudinal cracks develop on the specimen surface. One crack penetrates through the specimen, whereas the other remains a secondary tensile crack, and the crack paths are still dominated by axial propagation. The DIC results show that the high strain regions begin to concentrate around the cavities, although the strain localization bands remain relatively continuous. The cavities alter the local stress distribution and promote crack initiation around the cavity boundaries; nevertheless, the overall structural continuity has not yet been substantially weakened, and the specimen still exhibits tensile splitting dominated failure. When the dissolution degree reaches 10%, the dominant crack becomes curved and branched, and its propagation path is deflected by the spatial distribution of the cavities. Local shear cracks develop near the bottom of the specimen and are accompanied by block dislocation and frictional sliding. The high strain regions become more dispersed, with localized concentrations developing around the dominant crack. Accordingly, the cracking mode gradually transforms from single tensile crack coalescence to the combined action of tensile and shear cracking. At a dissolution degree of 15%, the crack morphology becomes increasingly complex. Multiple longitudinal and branched cracks develop on the specimen surface, and the dominant and secondary cracks jointly form coalesced failure paths. Crack initiation becomes more spatially dispersed, and the propagation paths are more strongly controlled by the cavities. The high strain regions exhibit multiple localized concentrations and gradually form band shaped distributions, while local shear bands progressively develop. Cracks not only propagate along the cavity boundaries but also coalesce, deflect, and slide within the weakened regions between adjacent cavities, exhibiting coordinated propagation controlled by multiple defects. At a dissolution degree of 20%, the number of cracks increases considerably, and their spatial distribution becomes more strongly controlled by the cavities. In addition to axial cracks, distinct inclined shear cracks are observed. The high strain regions are heterogeneously distributed and extend over a larger area, indicating that the effective load bearing skeleton has been segmented by the cavities into multiple weakened regions. Cracks can therefore initiate prematurely around several cavity boundaries and propagate and coalesce along the weakened zones. Meanwhile, block dislocation and frictional sliding become more pronounced, and the shear contribution increases, resulting in distinct progressive damage and mixed tensile-shear failure.

It should be noted that the rock-like specimens with different dissolution degrees used in this study represent simplified physical models of naturally dissolved rock masses. The experimental results are intended to reveal the mechanisms by which dissolution degree affects crack evolution and mechanical failure characteristics under uniaxial compression. Therefore, the following engineering discussion should be regarded as qualitative implications rather than direct design criteria. For underground engineering applications such as tunnels, the dissolution degree dependent failure characteristics provide important implications for support design. Surrounding rock with a low dissolution degree is generally dominated by overall tensile splitting failure after excavation induced unloading and exposure of the excavation surface. In such cases, conventional shotcrete and rock bolt support can be employed to restrain the opening of macroscopic tensile cracks. For surrounding rock with a high dissolution degree, pronounced local tensile stress concentrations may still develop within densely distributed dissolution defect regions, even when the overall rock mass retains relatively satisfactory integrity. Cracks can readily initiate and propagate around the dissolution defects, potentially inducing local block detachment and surface spalling. Therefore, advanced geological prospecting, grouting based cavity filling and reinforcement, and timely closure of the support system should be adopted to restrain the development of tensile cracks around the cavities and mitigate the risk of localized tensile instability.

## 4. Conclusions

In this study, a preparation method for rock-like specimens with dissolution defects was developed, and a mechanical-acoustic-optical integrated experimental system was established to characterize their failure processes under uniaxial compression. The mechanical, acoustic, and optical characteristics of specimens with different dissolution degrees were systematically analyzed, and the effects of dissolution degree on crack initiation, propagation, and macroscopic failure modes were revealed. The main conclusions are as follows:

(1) Increasing dissolution degree significantly weakens the load bearing capacity and deformation resistance of the rock-like specimens. As the dissolution degree increases from 0% to 20%, the compressive strength, elastic modulus, peak axial strain, and deformation modulus decrease by 36.45%, 27.01%, 17.25%, and 41.28%, respectively. Cracks initiate and propagate at lower stress levels, while the post-peak failure behavior transforms from the rapid coalescence of a single dominant crack to progressive failure controlled by multiple defects.
(2) The AE ring counts exhibit satisfactory correspondence with the stress evolution and DIC axial strain fields. With increasing dissolution degree, AE activity gradually changes from concentrated release near the peak strength to continuous release throughout the loading process. The onset of active AE behavior occurs earlier, the pre-peak damage accumulation interval expands, and the crack initiation threshold decreases. Consequently, the failure behavior progressively transforms from concentrated brittle failure to progressive damage and failure.
(3) As the dissolution degree increases, the proportion of high frequency signals decreases continuously, whereas the proportions of intermediate and low frequency signals increase markedly. Crack activity consequently evolves from microcrack initiation and propagation to the coalescence and through going propagation of larger scale cracks, accompanied by enhanced frictional sliding. The proportion of tensile cracks decreases from 61.81% to 42.74%, while the proportion of shear cracks increases correspondingly. The cracking mode therefore gradually evolves from tensile dominated cracking to mixed tensile-shear cracking with an increasingly pronounced shear contribution.
(4) The DIC shear strain fields and macroscopic failure morphologies demonstrate that dissolution degree exerts a pronounced controlling effect on crack propagation paths and failure modes. With increasing dissolution degree, the specimens progressively evolve from axial tensile splitting failure characterized by a limited distribution of shear strain zones and a relatively simple crack path to progressive mixed tensile-shear failure characterized by an increasing number of shear strain concentration zones, crack initiation at multiple locations, and the coordinated coalescence of multiple cracks.
